# Production and Characterization of Cross-Linked Aggregates of *Geobacillus thermoleovorans* CCR11 Thermoalkaliphilic Recombinant Lipase

**DOI:** 10.3390/molecules26247569

**Published:** 2021-12-14

**Authors:** Rosa-María Oliart-Ros, Giselle-Lilian Badillo-Zeferino, Rodolfo Quintana-Castro, Irving-Israel Ruíz-López, Alfonso Alexander-Aguilera, Jorge-Guillermo Domínguez-Chávez, Azmat Ali Khan, Dinh Duc Nguyen, Ashok Kumar Nadda, María-Guadalupe Sánchez-Otero

**Affiliations:** 1 Unidad de Investigación y Desarrollo en Alimentos, Tecnológico Nacional de México, Instituto Tecnológico de Veracruz, M.A. De Quevedo 2779, Veracruz C.P. 91897, Ver., Mexico; rosa.or@veracruz.tecnm.edu.mx (R.-M.O.-R.); gbadizef@gmail.com (G.-L.B.-Z.); 2 Facultad de Bioanálisis, Universidad Veracruzana, Carmen Serdán Esq. Iturbide, Veracruz C.P. 91700, Ver., Mexico; roquintana@uv.mx (R.Q.-C.); aalexander@uv.mx (A.A.-A.); jorgedominguez@uv.mx (J.-G.D.-C.); 3 Facultad de Ingeniería Química, Benemérita Universidad Autónoma de Puebla, Av. San Claudio y 18 Sur, Ciudad Universitaria, Puebla C.P. 72570, Pue., Mexico; irving.ruiz@correo.buap.mx; 4 Pharmaceutical Biotechnology Laboratory, Department of Pharmaceutical Chemistry, College of Pharmacy, King Saud University, Riyadh 11451, Saudi Arabia; azkhan@ksu.edu.sa; 5 Department of Environmental and Energy Engineering, Kyonggi University, 154-42 Gwanggyosan-ro, Yeongtong-gu, Suwon-si 16227, Gyeonggi-do, Korea; dinhduc.nguyen@kyonggi.ac.kr; 6 Faculty of Environmental and Food Engineering, Nguyen Tat Thanh University, 300A Nguyen Tat Thanh, District 4, Ho Chi Minh City 755414, Vietnam; 7 Department of Biotechnology and Bioinformatics, Faculty of Biotechnology, Jaypee University of Information Technology, Waknaghat, Solan, Himachal Pradesh 173 234, India

**Keywords:** cross-linked enzymatic aggregates (CLEAs), thermoalkaliphilic lipase, *Geobacillus thermoleovorans*

## Abstract

Immobilization of enzymes has many advantages for their application in biotechnological processes. In particular, the cross-linked enzyme aggregates (CLEAs) allow the production of solid biocatalysts with a high enzymatic loading and the advantage of obtaining derivatives with high stability at low cost. The purpose of this study was to produce cross-linked enzymatic aggregates (CLEAs) of LipMatCCR11, a 43 kDa recombinant solvent-tolerant thermoalkaliphilic lipase from *Geobacillus thermoleovorans* CCR11. LipMatCCR11-CLEAs were prepared using (NH_4_)_2_SO_4_ (40% *w*/*v*) as precipitant agent and glutaraldehyde (40 mM) as cross-linker, at pH 9, 20 °C. A U_10_(5^6^) uniform design was used to optimize CLEA production, varying protein concentration, ammonium sulfate %, pH, glutaraldehyde concentration, temperature, and incubation time. The synthesized CLEAs were also analyzed using scanning electron microscopy (SEM) that showed individual particles of <1 µm grouped to form a superstructure. The cross-linked aggregates showed a maximum mass activity of 7750 U/g at 40 °C and pH 8 and retained more than 20% activity at 100 °C. Greater thermostability, resistance to alkaline conditions and the presence of organic solvents, and better durability during storage were observed for LipMatCCR11-CLEAs in comparison with the soluble enzyme. LipMatCCR11-CLEAs presented good reusability by conserving 40% of their initial activity after 9 cycles of reuse.

## 1. Introduction

Lipases (E.C. 3.1.1.3) are ubiquitous enzymes, as they have been found to occur in most living organisms; in vivo, they catalyze the hydrolysis of triacylglycerides to glycerol and fatty acids [1]. In non-aqueous media, lipases can synthesize a wide range of esters or compounds with analogous bonding as amides; due to these features, lipases are widely applied in many industrial areas, such as the food industry, the production of biopharmaceuticals, biofuels, biopolymers, and detergents [2,3,4,5].

Lipases share common structural features such as the canonical α/β hydrolase fold, the catalytic triad, and the oxyanion hole [6,7]. Some of them possess a flexible alpha-helix (called “lid”) covering their active site, which in its ‘‘closed conformation’’ makes it inaccessible to substrates; this form exists in equilibrium with an “open form”. However, in the presence of hydrophobic interfaces, the equilibrium between the open and closed forms shifts to the “open form” through important conformational changes, where the active site becomes accessible and hydrophobic surfaces are exposed, increasing enzyme activity, a phenomenon called interfacial activation. This phenomenon is very important for all applications of lipases [8,9]. Thermoalkaliphilic lipases are particularly interesting due to their high stability towards high temperatures and pH, chaotropic agents, detergents, and some protease activity [1,6,10].

Immobilization of enzymes has many advantages since it allows the recovery and reuse of biocatalysts, improves reaction control, and generally has a positive impact on thermal stability as well as the stability in the presence of organic solvents and other chaotropic agents, and can improve the resistance to drastic pH values; immobilization also facilitates the separation of products and avoids contamination by the enzyme; therefore, immobilization is a key factor in the establishment of novel lipase applications and expansion of the current ones. Since there are many factors that are involved to achieve a successful immobilization of an enzyme, it is necessary to explore different methods in order to improve the enzyme stability, specificity, selectivity, and general performance [11,12,13,14,15,16,17].

In the traditional methods of immobilization, a solid support is used to bind the enzyme, which provides mechanical resistance and protection from direct heat shock [11,12,13,14,15,17,18,19,20]. In this context, the production of cross-linked enzyme aggregates (CLEAs) has proven to be an important immobilization technique that allows the production of solid biocatalysts with a high enzymatic loading and the advantage of obtaining derivatives with higher stability than their soluble counterparts and reducing costs, as there are no carrier expenses [20,21,22]. CLEA preparation might be achieved using different precipitants and cross-linkers; although it is supposed to be a simple technology, there are many experimental factors involved that may affect the particle size, stability, and catalytic capabilities of the final product; also, the size and solubility of the substrates might not be fully compatible with the pore size of resultant CLEAs, decreasing the activity since only the enzymes on the surface of the aggregate are catalytically active [16,20]. The synthesis of CLEAs involves the covalent cross-linking of a precipitated enzyme; the precipitation is achieved by adding agents such as polymers, inorganic salts, and organic solvents; among them, inorganic salts and polymers are preferred for CLEA synthesis as they promote the formation of aggregates without affecting the three-dimensional structure of the proteins [23,24]. To fix the network permanently after precipitation, intermolecular unions are formed by using bifunctional reagents, such as diiminoesters, dihydrins and diamines [20,25], and most recently, sodium tripolyphosphate [26]. In this regard, the most used cross-linking agent is glutaraldehyde, which can react with several functional groups of proteins, including thiol, phenol, and imidazole, but preferably with the ε-amino groups found, for example, in the side group of lysines. In aqueous medium, glutaraldehyde has a strong tendency to self-polymerize, depending on its concentration and medium pH. Alkaline conditions promote self-polymerization, while acidic conditions decrease self-polymerization and enhance glutaraldehyde stability. The mechanism is generally not limited to a single reaction, since it depends on different trigger points such as polymerization products or reaction products with different functional groups [27]; ideally the lysines located in the surface of the enzyme react with one of the aldehyde extremes, giving rise to a first Schiff base; then, the free aldehyde group can establish a covalent bond with another enzyme molecule [25]. The result of the reticulation process is a network of enzymes with irreversible intermolecular bonds capable of withstanding harsh conditions of pH and temperature, although the reaction might be incomplete at the interior of the aggregate, affecting the final stability of the solid enzyme [20,25,27]. Since the initial step of precipitation could be considered a form of purification, the process can be efficiently used with cell lysates or fermentation broths [20,28].

One of the most important objectives of an enzyme′s immobilization is to improve its storage and reusability, which provides opportunities for several cycles of biocatalysis. Some authors have addressed these properties in CLEAs of different enzymes such as a serine hydroxyl methyltransferase from *Idiomerina leihiensis* [12], a lipase from *Rhizopus oryzae* [29], or a lipase from *Candida rugosa* [30]. Reusability assays have demonstrated around 10 cycles of use, followed by a loss of activity due to mechanical forces and leaching or to denaturation of the enzyme [24]

Regarding the synthesis of CLEAs of lipolytic enzymes, the literature includes agents such as *tert*-butyl alcohol (50–90% *v*/*v*) and ammonium sulfate (20–60% saturation) among the more-used compounds for inducing precipitation [31]; in other hydrolases, other agents have been reported such as cooled acetone (100%) as an effective precipitant for a thermostable xylanase [22]. Other reaction conditions (precipitation time, pH, temperature, agitation, incubation time, protein source, use of additives) can also be modified to obtain a product with the desired characteristics [23,24]; therefore, the study of the cross-linking conditions is a mandatory step in the synthesis of CLEAs of any enzyme. With respect to the stability of CLEAs of lipases, there are reports of increased thermostability in CLEAs prepared with commercial lipases such as *Candida antarctica* lipase B (CALB-L), *Thermomyces lanuginosa* lipase, *C. rugosa* lipase, and *Rhizopus delemar* lipase using PEGs and acetone as precipitants [32].

In a previous study, the thermoalkaliphilic lipase LipMatCCR11 from *Geobacillus thermoleovorans* CCR11 was expressed in *Escherichia coli* [33]. A 43 kDa enzyme, LipMatCCR11 has 11 lysine residues in its structure, none of which is directly involved with the active site or the domains of interaction with Ca^+2^ and Zn^+2^, two of the Lys residues located in the lid region. In a previous study, LipMatCCR11 was used in lyophilized form for the synthesis of flavors and fragrance esters with hexane as a reaction medium [3]. Later, using response surface methodology, conditions for maximum expression were investigated and a 1300-fold increase in lipolytic activity was achieved; this enzyme was also immobilized in Accurel EP-100 and used to synthetize aromatic esters [34]. The aims of the present work were to establish the best conditions to produce cross-linked aggregates of the recombinant thermoalkaliphilic lipase LipMatCCR1 using ammonium sulfate as precipitation agent and glutaraldehyde as cross-linking agent, and to characterize the immobilized product.

## 2. Results and Discussion

### 2.1. Optimization of LipMatCCR11-CLEA Production

**Figure 1:**
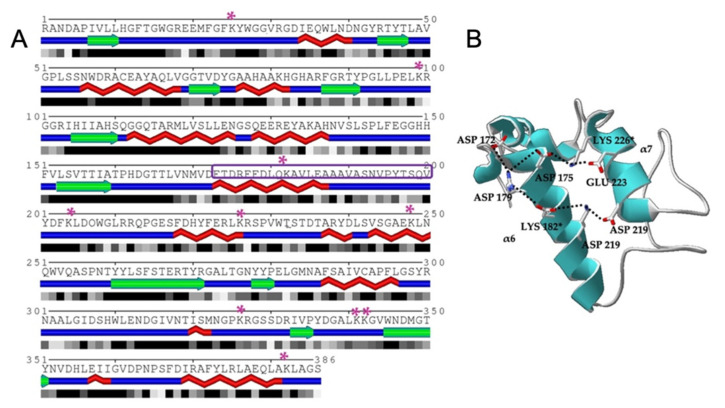
(**A**) Expected secondary structures based on the amino acid sequence of the LipMatCCR11 lipase are depicted; Lys residues are marked with a purple * and lid sequence is delimited by a purple rectangle (model generated in http://sable.cchmc.org/, accessed on 23 October 2021) [35]. (**B**) LipMATCCR11 Lid domain generated with MODELLER v1.11 tool [36]; Lys 182 and Lys 226 participate in the lid opening.

For LipMatCCR11-CLEA preparation, glutaraldehyde was selected as the reticulant agent since LipMatCCR11 has 11 lysine residues in its structure, none of which is directly involved in the active site or the domains of interaction with Ca^+2^ and Zn^+2^ (Figure 1A). By comparison with the *Geobacillus thermocatenulatus* lipase [6], it was assumed that the residues Lys182 and Lys226 in LipMatCCR11 might be involved in the salt bridges that are destabilized when the lid is opened, and in consequence, could participate in the cross-linking process with aldehyde groups, promoting an artificial opening of the lid (Figure 1B).

Optimization of the synthesis of CLEAs using experimental design strategies and statistical data management is a relatively recent field of study [28]. The most frequently used experimental designs for this purpose are full factorial designs, which are robust and straightforward in approach; however, with this type of design, a limited number of variables and levels can be considered [24,37,38,39]. In the present work, a U_10_(5^6^) uniform design allowed us to assess the effect of 6 factors in 5 levels.

LipMatCCR11 lipase crude extract was obtained as the supernatant after the centrifugation of a sonicated fresh culture of the recombinant *E. coli*-pET-3b-LipMatCCR11 as explained in Section 3.1. Prior to its use for each treatment, the enzymatic crude extract was characterized with respect to its protein content and lipolytic activity. The protein concentration in the crude extract was 28 ± 2 mg/mL, and the volumetric lipolytic activity was 2547 ± 133 U/mL with a specific activity of 91 ± 4 U/mg.

**Figure 2:**
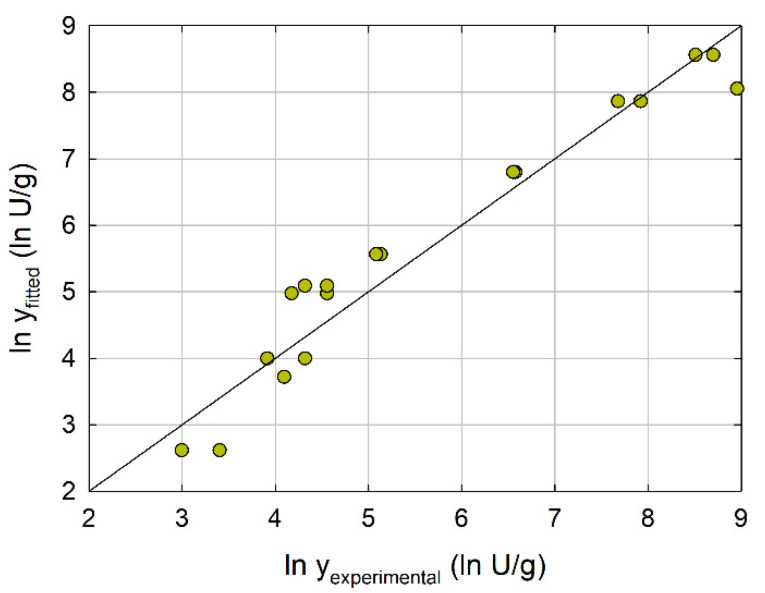
Linear regression of the model obtained in optimization of LipMatCCR11-CLEA production.

Stepwise regression allowed the identification of the response equation, where each *x_j_* (*j* = 1, …, 5) represented the coded factor level (Equation (1)). This model demonstrated an excellent reproduction of the experimental behavior by having a coefficient of determination higher than 0.95, which indicated that no more than 5% of the total variance in the data could not be explained by the regression (Figure 2). According to this model, glutaraldehyde concentration (×4) was the variable showing the highest influence (negative) on CLEA activity, while the effect of the other factors was similar. In this case, higher CLEA activities were associated with increasing protein concentration (×1) and pH levels (×3), while precipitation agent (×2) and temperature (×5) had the reverse effect.

(1) $$\ln y=5.72+0.54x_{1}-0.62x_{2}+0.51x_{3}-2.44x_{4}-0.69x_{5}\;(R^{2}=0.95)$$

Significant variables were analyzed using the path of steepest ascent; however, no further increase in the response variable beyond the maximum obtained during the experimental development of the design was observed. Therefore, the characterization was performed using the combination of studied variables corresponding to treatment 8, which were: a protein concentration of 3.25 mg/mL, 40% (NH_4_)_2_SO_4_ as the precipitant agent, pH 9, glutaraldehyde 40 mM as the cross-linker agent, reaction temperature of 20 °C, for 7.5 h.

The mass activity for the LipMatCCR11-CLEAs was 7750 U/g, and the amount of protein immobilized was 520 mg of protein/g. An important decrease in lipolytic activity was observed (85%, in comparison with soluble enzyme), which could be related to a change in the enzyme conformation during immobilization, and/or to an insufficient rigidity of the enzyme. The interference of the host´s proteins with the immobilization processes could be another factor affecting enzymatic activity. Nevertheless, the improvement in thermostability and the capacity for reuse (see below) give LipMatCCR11-CLEAs added value [17,40].

### 2.2. Biochemical Characterization

#### 2.2.1. Effect of Temperature and pH on LipMatCCR11-CLEA Activity

**Figure 3:**
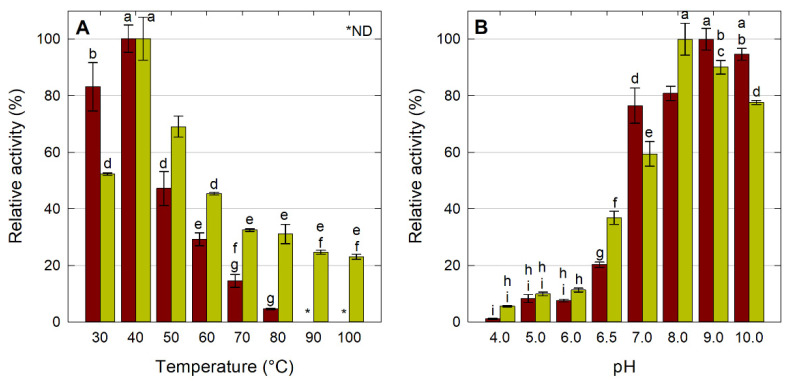
Characterization of LipMatCCR11-CLEAs. Effect of temperature (**A**) and pH (**B**) on lipolytic activity. All values are the mean of three replicates. Red: LipMatCCR11, Green: LipMatCCR11-CLEAs Error bars represent the standard deviation within the data set. Different lowercase letters indicate significant differences according to Tukey’s multiple comparison tests (*p* < 0.05). The 100% value corresponds to a CLEA activity of 7755 ± 144 U/g. *: Non-detectable.

The effect of temperature and pH on the hydrolytic activity of soluble enzyme and LipMatCCR11-CLEAs is depicted in Figure 3A. The optimal temperature was recorded at 40 °C for both the soluble enzyme and the CLEAs.

It was found that while a sharp decrease in activity was observed in the soluble enzyme at temperatures above 70 °C, more than 20% of the activity of LipMatCCR11-CLEAs was conserved at over 100 °C; this phenomenon has been observed with CLEAs for other enzymes, and it is attributed to a decrease in the conformational flexibility of the enzyme associated with the formation of covalent bonds within the aggregates [28,29,38].

The effect of pH on the hydrolytic activity was studied at pH 4–10 at 40 °C; higher pH values were not assessed due to the non-enzymatic hydrolysis of the substrate (*p*-nitrophenyl-laurate). The activity profiles obtained are represented in Figure 3B. The optimal pH values for the soluble and immobilized enzyme were 9 and 8, respectively. There are reports in the literature of similar variations in the optimal pH between soluble and immobilized enzymes by this method; although the CLEA formation process does not involve the use of any support, previous studies have reported changes in the optimal pH of different hydrolases that can tend towards alkalinity [22,29] or acidity [24,31]. Regarding the change to more acidic pH values, Mahmod et al. [24] observed a change in the optimal pH value of the free enzyme from 8 to 6.8 when the CLEAs were synthesized with the *Ictalurus punctatus* protease.

**Figure 4:**
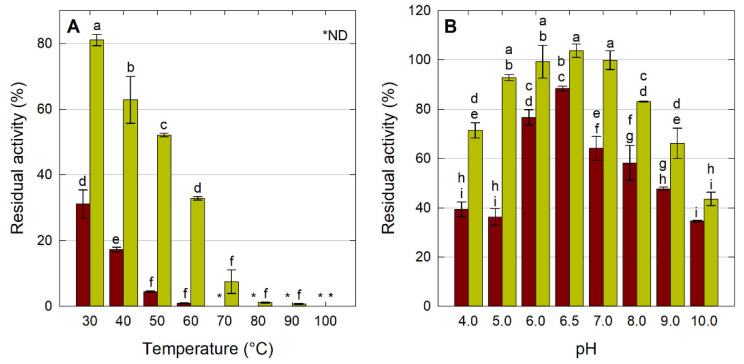
Characterization of LipMatCCR11-CLEAs. Effect of temperature (**A**) and pH (**B**) on lipolytic stability. All values are the mean of three replicates. Red: LipMatCCR1, Green: LipMatCCR11-CLEAs. Error bars represent the standard deviation within the data set. Different lowercase letters indicate significant differences according to Tukey's multiple comparison tests (*p* < 0.05). The 100% value corresponds to a CLEA activity of 7755 ± 144 U/g. *: Non-detectable.

Figure 4A shows the results from the thermostability assay; the soluble enzyme lost more than 70% of its activity after 1 h at 30 °C and only conserved 1% of its activity at 60 °C, while the LipMatCCR11-CLEAs conserved more than 30% of their residual activity under the same conditions. Similarly, increases in the thermostability of CLEAs have been reported for *Rhizopus orizae* lipase, whose cross-linked derivatives retained 50% of their activity after incubation at 60 °C, in contrast to its free form in which activity was completely lost after being incubated for 15 min at the same temperature [29].

The effect of pH on the stability of the soluble LipMatCCR11 and LipMatCCR11CLEAs was evaluated by quantifying the residual activity after incubating the enzyme preparations for 24 h at room temperature in buffers of pH 4–10. The percentages of residual activity of the soluble and immobilized lipase are presented in Figure 4B. The LipMatCCR11-CLEAs exhibited a stability that was significantly higher (10–50%) than the soluble enzyme in the evaluated pH range. The increase in stability at different pHs for immobilized hydrolases has been previously described, and it has been attributed to the protection that the cross-linked network provides to the reacting groups of the enzyme structure [23]. The fact that pH resistance was lower at optimum pH in comparison with neutral pHs could be due to changes in the protonation state of some functional groups as a result of the prolonged exposure of the enzyme to the alkaline environment [41].

#### 2.2.2. Effect of Metal Ions on LipMatCCR11-CLEA Activity

**Figure 5:**
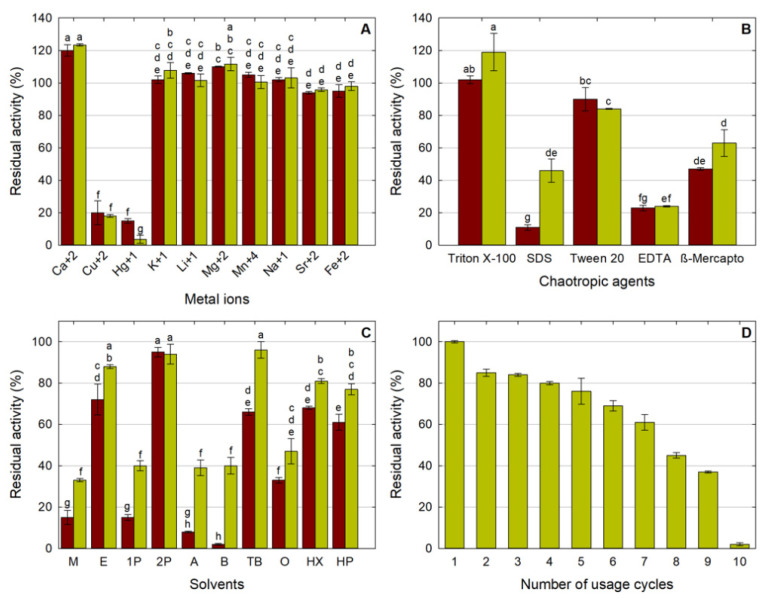
Characterization of LipMatCCR11-CLEAs. (**A**) Stability against metal ions; (**B**) stability on chaotropic agents; (**C**) stability on solvents M: methanol, E: ethanol, 1P: 1-propanol, 2P: 2-propanol, A: acetone, B: butanol, TB: *tert*-butanol, O: octanol, HX: hexane, HP: heptane. (**D**) Reusability of LipMAtCCR11-CLEAs. All values are the mean of three replicates Red: LipMatCCR1, Green: LipMatCCR11-CLEAs. Error bars represent the standard deviation within the data set. Different lowercase letters indicate significant differences according to Tukey’s multiple comparison tests (*p* < 0.05). The 100% value corresponds to a CLEA activity of 7755 ± 144 U/g.

The residual activity of the soluble lipase and the LipMatCCR1-CLEAs after their incubation in solutions of different inorganic chlorides is depicted in Figure 5A. As can be seen, there were no significant differences (*p* < 0.05) in the lipolytic activity of CLEAs and soluble lipase due to exposure to metal ions except for Ca^+2^, Cu^+2^, and Hg^+1^; exposure to Ca^+2^ provoked a significant activation (120%) in LipMatCCR11-CLEA activity that could be due to the promotion of conformational changes of the enzyme in domains associated with thermostability [31]. The increase in lipolytic activity after incubation in the presence of some divalent cations has been reported for lipases and esterases from *Bacillus* and *Geobacillus* [42,43,44]. Cu^+2^ and Hg^+1^ provoked an inhibitory effect of 80 and 95%, respectively, on both forms of the enzyme. A similar effect has been reported for other thermophilic lipases and esterases [43,45], and although its exact role in the inactivation of lipases has not been clarified, this phenomenon could be due to the induction of a structural disturbance in the protein, probably due to the association with residues that participate in the catalytic processes [46].

#### 2.2.3. Stability in Chaotropic Agents

As depicted in Figure 5B, there were no significant differences between the activities of LipMatCCR11-CLEAs and their soluble counterparts in the presence of Triton X-100, Tween 20, and β-mercaptoethanol (*p* < 0.05). In contrast, sodium dodecyl sulfate (SDS) provoked a 90% decrease in the activity of the soluble enzyme, while LipMatCCR11-CLEAs retained more than 40% of their residual activity under the same conditions. A similar behavior was reported for the bran grain lipase after its immobilization by entrapment in alginate beads, which presented a 50% increase in its resistance to SDS-mediated inactivation [47]. Nonionic surfactants such as Triton X-100 or Tween^®^ series compounds are considered "softer" than their charged counterparts, and usually have an activating effect on lipases because they can emulate the chemical environment favorable to triggering the active catalytic conformation [48]. As observed in the lipase obtained from *Geobacillus* sp. EPT9 [49], Tween^®^ 20 caused a moderate inhibition of LipMatCCR11 in its two forms. Reducing agents such as β-mercaptoethanol mainly affect the cysteine residues that form disulfide bridges; LipMatCCR11 lipase contains only two cysteine residues in its sequence, and the moderate inactivation caused by β-mercaptoethanol for both forms of the enzyme suggests that these residues are not a part of catalytic site stabilization. After treatment with EDTA, LipMatCCR11-CLEAs lost more than 70% of their initial activity, so it can be inferred that the cross-linking was not able to fix the active conformation induced by the presence of cations usually associated with stability, such as Ca^+2^ [50].

#### 2.2.4. Stability in Organic Solvents

Stability in organic solvents was evaluated using 10 solvents with different log P. The residual activity after each treatment is depicted in Figure 5C. In general, LipMatCCR11-CLEAs were significantly (*p* < 0.05) more stable than the soluble lipase in the presence of all the solvents studied; the immobilized variety retained 2 to 20 times more activity than the soluble enzyme in polar solvents such as methanol, 1-propanol, acetone, and *n*-butanol. Despite the resistance of lipases to the effect of hydrophobic organic solvents [51], the behavior that these enzymes show against solvents considered polar (log *p* < 2.0) is usually variable [52].

Although there has been an increase in the implementation of green chemistry-based protocols, the use of polar solvents continues as part of the reaction medium or as building blocks; the use of short-chain alcohols to produce biodiesel is an example of both applications [53]. Detailed experiments regarding the effect of different organic solvents on the catalytic activity of CLEAs have shown that the cross-linking process increases the stability against polar compounds, especially against short-chain alcohols. This high stability can be attributed to the fact that the immobilized enzyme structure is stabilized by cross-linking and manages to maintain its catalytic activity even after the removal of the solvation layer from the enzyme surface caused by solvents [54,55].

#### 2.2.5. Reusability of Immobilized Lipase Test

For any industrial application, the possibility of reusing the enzymes is of vital importance to the goal of reducing total production costs [53,56]. As can be seen in Figure 5D, after being subjected to nine cycles under experimental conditions, LipMatCCR11-CLEAs retained 37% of their initial activity and reached total inactivation after 10 cycles. A similar reusability was reported by Jamwal et al. [57] for the cross-linked aggregates of the thermostable lipase from *Geobacillus* sp. CLEA reuse capacity may vary due to multiple factors such as the nature of the enzyme used, the production process, or the test conditions; the loss of activity during reuse might be due to changes in CLEA morphology as a result of mechanical agitation, or to the leaching of the enzymes into the reaction medium [23,30,31,39].

#### 2.2.6. Storage Stability of LipMatCCR1-CLEAs

Storage at 4 °C and 25 °C for 30 days did not exert a significant effect on the activity of the LipMatCCR11-CLEAs (*p* < 0.05). These results agree with the experiments conducted by Yang et al. [58], in which CLEAs of *Thermomyces lanuginosus* lipase did not show any significant decrease in activity after 20 days of storage at 4 °C. Other CLEAs have been reported with outstanding storage stability, such as the common mushroom tyrosinase CLEAs stored at 4 °C and 25 °C for 3 months, retaining 84 and 83% of their activity, respectively [28].

#### 2.2.7. Morphological Analysis

**Figure 6:**
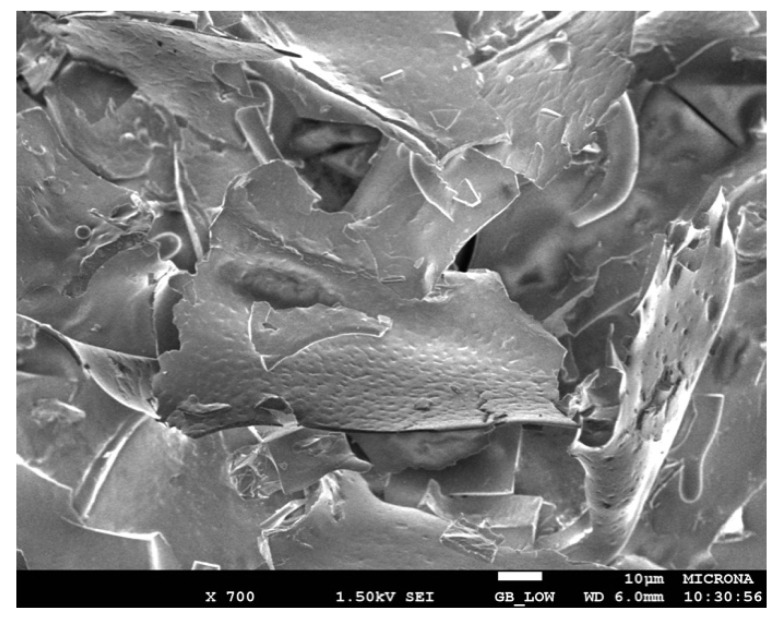
SEM Image of LipMatCCR11 in lyophilized form. Magnification 700×.

The morphology and size of the LipMatCCR11-CLEAs and the lyophilized soluble protein were determined by scanning electron microscopy. In the lyophilized sample, large lamellar structures of irregular shape were observed (Figure 6), similar to what was observed in reported micrographs of other protein lyophilizates, which in general can be attributed to the fragile nature of the resulting solid [59].

**Figure 7:**
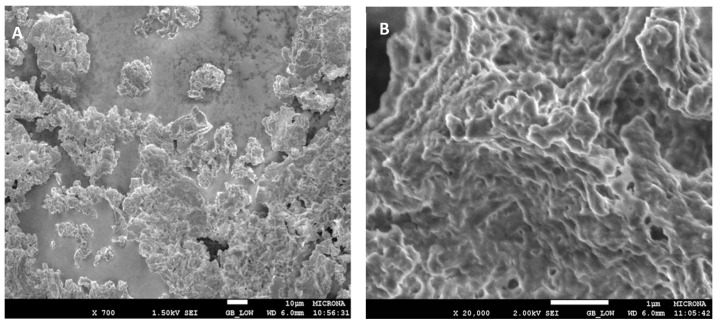
SEM Image of LipMatCCR11-CLEAs. (**A**) Magnification: 700×. (**B**) Magnification: 20,000×.

Regarding the morphology of the LipMatCCR11-CLEAs, Figure 7 shows individual aggregates of approximately 0.25 µm in diameter that form clusters or branched aggregates with sizes ranging from 10 to 100 µm. This places them in the group of type 2 CLEAs (associated aggregates in large groups with individual sizes of less than 1 µm). The formation of branched aggregates could be the consequence of covalent bond formations between the reactive groups on the surfaces of the individual aggregates.

## 3. Materials and Methods

Reagents, solvents, and culture media were of reagent grade, used without any additional purification, and they were obtained from Sigma-Aldrich Química, Toluca, México. To produce the lipase, *E. coli* BL21 (DE3) transformed with the plasmid pET-3b-LipMatCCR11 was used (*E. coli* BL 21 (DE3) was purchased from New England Biolabs, Ipswich, MA, USA; plasmid was purchased from Merck, Darmstadt, Germany) [34].

### 3.1. Production and Recovery of the LipMatCCR11 Lipase

LipMatCCR11 lipase was obtained from a fresh culture of the recombinant *E. coli*-pET-3b-LipMatCCR11 as described by Badillo-Zeferino et al. [34] as follows: After centrifugation cells were placed on ice and suspended in 0.1 M potassium phosphate buffer (pH 9). With the aid of an ultrasonic processor (Cole Palmer Ultrasonic Processor Mod. GB 130PB, Court Vernon Hills, IL, USA) cells were disrupted with ten 15 s pulses (total applied energy: 2500 J) separated by a 30 s pause, at a 20 kHz frequency and high amplitude (60%). Lipolytic activity and protein concentration were measured in the supernatant after centrifuging at 14,000× *g* at 4 °C for 15 min.

### 3.2. Lipolytic Activity and Protein Concentration

Lipolytic activity of both soluble and immobilized enzymes was determined by a spectrophotometric method: 5 mg of CLEA lipase or 100 μL of the diluted crude enzyme were incubated in 0.05 M phosphate buffer (pH 6.5; 0.9 mL final volume), and 0.1 mL of 10 mM *p*-nitrophenyl-laurate in ethanol for 30 min at 60 °C. Reaction was stopped with 250 mL of Na_2_CO_3_ and centrifugation at 16,000× *g* at 4 °C for 10 min, after which aliquots of the supernatants were taken and their absorbance at 405 nm measured in a Stat Fax^®^ 2100 microplate reader (Awareness Technologies, Bellport, NY, USA) [60]. The protein concentration was evaluated by the Lowry method [61].

### 3.3. Synthesis of the LipMatCCR11-CLEAs

*E. coli*-pET-3b-LipMatCCR11 was lysed and diluted with buffers of various pHs (6.5–9) in order to adjust the desired concentration of protein, as reported by López-Serrano et al. [62]. Then, Triton X-100 was added up to 0.15% *v*/*v*; next, ammonium sulfate was added up to each percentage (20–60) of final saturation. Immediately afterwards, the cross-linking process started with the addition of 25% aqueous glutaraldehyde solution. The reaction was allowed to continue under isothermal conditions at each selected temperature (4–25 °C), and once the time of each treatment was completed (2–24 h), reaction was stopped by adding buffer with the corresponding pH to dilute the saturation of ammonium sulfate below 15% in the reaction mixture. The LipMatCCR11-CLEAs were washed three times with the corresponding pH buffer in a buffer-CLEA ratio of 10:1, recovered by centrifugation at 9000× *g*, 15 min, 4 °C, and dried in a Speed Vacuum Concentrator Thermo Fisher Scientific^®^ (Asheville, NC, USA). CLEAs were stored at 4 °C.

### 3.4. Optimization of LipMatCCR11-CLEA Production

Six factors were selected in this study to investigate the production of the LipMatCCR11-CLEAs: (1) protein concentration (×1, mg protein/mL), (2) precipitation agent (×2, ammonium sulfate, % sat), (3) pH (×3), (4) glutaraldehyde concentration (×4, mM), (5) temperature (°C), and (6) incubation time. Each factor was evaluated at five levels (Table 1) according to a uniform design to better characterize the experimental region with a minimum number of experiments [63]. The selected U_10_(5^6^) uniform design (10 treatments with 6 factors at 5 levels) is presented in Table 2. Each treatment was carried out in duplicate.

Stepwise regression was applied to choose the predictive variables in the response equations. Besides only including significant terms, the final model was selected to have the highest *R*^2^ value and the lowest PRESS statistic. Linear regression (based on ordinary least squares), analysis of variance, and numerical procedures were performed with the Matlab software and its Statistic Toolbox 7.3 (Matlab R2010a, MathWorks Inc., Natick, MA, USA).

### 3.5. Characterization of the LipMatCCR11-CLEAs

#### 3.5.1. Effect of pH and Temperature on Lipase Activity

The optimum pH and temperature were determined by measuring the hydrolytic activity of soluble and immobilized enzymes in a pH range of 4–10 at 40 °C, and a temperature range of 30–100 °C, at pH of 6.5. The tests were carried out with 100 µL of enzymatic extract diluted in the corresponding buffer, or with 0.005 g of LipMatCCR11-CLEAs. Lipolytic activity was expressed as relative activity (%) [64].

#### 3.5.2. Effect of pH and Temperature on Enzyme Stability

Samples of both soluble and immobilized enzymes were incubated at different temperatures (30–100 °C) for 1 h. The residual activities were determined as described above. To determine the stability against exposure to different pH conditions, aliquots of 100 µL of enzymatic extract (5 mg protein/mL) and LipMatCCR11-CLEAs (approximately 0.050 g) were incubated for 24 h at 25 °C in 1 mL of buffer solution (50 mM) of different pHs: (4.0, 5.0, 6.0, 6.5, 7.0, 8.0, 9.0, and 10.0). After incubation, LipMatCCR11-CLEAs were removed by filtration and dried under vacuum; subsequently, residual lipolytic activity was determined under the conditions previously indicated at 40 °C. Results were expressed as residual activity (%).

#### 3.5.3. Effect of Metallic Ions and Chaotropic Agents on Enzyme Stability

In order to assess the effect of metal ions on the activity of soluble and LipMatCCR1-CLEAs, aliquots of 100 µL of enzyme extract (5 mg protein/mL) or 0.010 g of LipMatCCR11-CLEAs were incubated in a 1mM aqueous solution of CaCl_2_, KCl, MgCl_2_, NaCl, HgCl, LiCl, FeCl_2_, MgCl_2_, SrCl_2_, and CuCl_2_, and in solutions of detergents and chaotropic agents, all of them dissolved in 50 mM sodium phosphate buffer, pH 6.5. The compounds analyzed were the following: EDTA (1 mM), β-mercaptoethanol 1% (*v*/*v*), Triton X-100 1% (*v*/*v*), Tween^®^20 1% (*v*/*v*), and dodecyl sulfate of sodium (SDS) 0.1% (*w*/*v*). The incubation was carried out for 1 h at 30 ° C. At the end of incubation time, lipolytic activity was determined according to the standard conditions previously indicated, at 40 °C. Lipolytic activity was expressed as residual activity (%).

#### 3.5.4. Effect of Organic Solvents on Lipase Activity

To determine the stability of LipMatCCR11-CLEAs in organic solvents, samples of 0.010 g of LipMatCCR11-CLEAs and aliquots of 100 µL of enzyme extract (5 mg protein/mL) were incubated for 30 min in 500 µL of different organic solvents (methanol, ethanol, acetone, 1-propanol, 2-propanol, butanol, *tert*-butanol, hexane, heptane, octanol). Subsequently, the lipolytic activity of the samples was determined according to the standard conditions previously indicated, at 40 °C. Lipolytic activity was expressed as residual activity.

#### 3.5.5. Reutilization of LipMatCCR11-CLEAs

The study of the stability of LipMatCCR11-CLEAs for reuse was carried out as follows: the initial lipolytic activity was determined as previously indicated, and once the first cycle of activity was concluded, the LipMatCCR11-CLEAs were washed three times with 50 mM sodium phosphate buffer, pH 6.5, recovered by filtration, and dried under vacuum at room temperature. Once dry, the procedure was repeated until the lipolytic activity approached zero. The residual lipolytic activity was expressed as a percentage.

### 3.6. Microstructural Analysis

The morphology and size of LipMatCCR11-CLEAs and the soluble protein in lyophilized form were determined by electron scanning micrographs using a JEOL scanning electron microscope (SEM) model JSM-7600F (JEOL, Tokyo, Japan) located at the Center for Research in Micro and Nanotechnology (MICRONA) (Universidad Veracruzana). Prior to their observation, the LipMatCCR11-CLEAs and the lyophilized enzyme were stored in a vacuum desiccator; the micrographs of both samples were made without any additional coating with an operating voltage between 1.0 and 2.0 keV in Gentle Beam mode.

## 4. Conclusions

The immobilization of the recombinant thermoalkaliphilic lipase LipMatCCR11 in the form of cross-linked aggregates (CLEAs) allowed us to obtain a robust biocatalyst in solid form, free of support, with greater thermostability, and reusable and resistant to storage.

The statistical approach utilized in this work was not sufficient to maximize the lipolytic activity of the CLEAs. To improve the stability and catalytic capacity of LipMatCCR11-CLEAs, it would be necessary to investigate the structural changes occurring in the protein during the immobilization process. These studies will be undertaken in the near future. To the best of our knowledge, this is the first report that describes the production of cross-linked aggregates of a thermoalkaliphilic lipase, their biochemical characterization, and the assessment of their reutilization capacity and storage resistance.

## Figures and Tables

**Table 1:** Factor and levels for investigating the production of the LipMatCCR11-CLEAs.

Factor	Code	Levels *				
		1 (−1)	2 (−1/2)	3 (0)	4 (1/2)	5 (1)
Protein concentration (mg/mL)	*x*_1_	1	3.25	5.5	7.75	10
Ammonium sulfate (% sat)	*x*_2_	20	30	40	50	60
pH	*x*_3_	6.5	7.125	7.75	8.375	9
Glutaraldehyde (mM)	*x*_4_	40	105	170	235	300
Temperature (°C)	*x*_5_	4	9.25	14.5	19.75	25
Incubation time (h)	*x*_6_	2	7.5	13	18.5	24

* Numbers in parentheses represent the coded levels used in the regression analysis.

**Table 2:** Production of the LipMatCCR11-CLEAs according to uniform design U_10_(5^6^).

Treatment	*x*_1_	*x*_2_	*x*_3_	*x*_4_	*x*_5_	*x*_6_	*y* (U/g) *
1	4	4	5	4	3	5	80 ± 22 ^e^
2	3	2	2	1	2	5	5485 ± 626 ^b^
3	5	4	3	2	1	1	2458 ± 344 ^c^
4	1	5	3	2	5	4	85 ± 31 ^e^
5	5	1	4	3	4	4	710 ± 12 ^d^
6	2	1	1	3	3	1	165 ± 6 ^de^
7	3	5	1	4	2	3	60 ± 0 ^e^
8	2	3	5	1	4	2	7755 ± 144 ^a^
9	1	2	4	5	1	3	63 ± 15 ^e^
10	4	3	2	5	5	2	25 ± 6 ^e^

* Values followed by a different letter are statistically different (Tukey’s test, *p* < 0.05).

## Data Availability

Not applicable.

## Sample Availability

Samples of the compounds are not available from the authors.
